# Sumoylation of HDAC2 promotes NF-κB-dependent gene expression

**DOI:** 10.18632/oncotarget.3344

**Published:** 2015-01-21

**Authors:** Tobias Wagner, Nicole Kiweler, Katharina Wolff, Shirley K. Knauer, André Brandl, Peter Hemmerich, Jan-Hermen Dannenberg, Thorsten Heinzel, Günter Schneider, Oliver H. Krämer

**Affiliations:** ^1^ Centre for Molecular Biomedicine, Institute of Biochemistry and Biophysics, Department of Biochemistry, Friedrich Schiller University of Jena, Jena, Germany; ^2^ Department of Toxicology, University Medical Center, Mainz, Germany; ^3^ Centre for Medical Biotechnology, Molecular Biology II, University of Duisburg-Essen, Essen, Germany; ^4^ Leibniz-Institute for Age Research, Fritz-Lipmann-Institute, Jena, Germany; ^5^ Division of Gene Regulation, Netherlands Cancer Institute, Amsterdam, The Netherlands; ^6^ Klinikum rechts der Isar, II. Medizinische Klinik, Technische Universität München, München, Germany

**Keywords:** Histone deacetylase 2, p53, p65, RSK1, SUMO

## Abstract

The transcription factor nuclear factor-κB (NF-κB) is crucial for the maintenance of homeostasis. It is incompletely understood how nuclear NF-κB and the crosstalk of NF-κB with other transcription factors are controlled. Here, we demonstrate that the epigenetic regulator histone deacetylase 2 (HDAC2) activates NF-κB in transformed and primary cells. This function depends on both, the catalytic activity and an intact HDAC2 sumoylation motif. Several mechanisms account for the induction of NF-κB through HDAC2. The expression of wild-type HDAC2 can increase the nuclear presence of NF-κB. In addition, the ribosomal S6 kinase 1 (RSK1) and the tumor suppressor p53 contribute to the regulation of NF-κB by HDAC2. Moreover, TP53 mRNA expression is positively regulated by wild-type HDAC2 but not by sumoylation-deficient HDAC2. Thus, sumoylation of HDAC2 integrates NF-κB signaling involving p53 and RSK1. Since HDAC2-dependent NF-κB activity protects colon cancer cells from genotoxic stress, our data also suggest that high HDAC2 levels, which are frequently found in tumors, are linked to chemoresistance. Accordingly, inhibitors of NF-κB and of the NF-κB/p53-regulated anti-apoptotic protein survivin significantly sensitize colon carcinoma cells expressing wild-type HDAC2 to apoptosis induced by the genotoxin doxorubicin. Hence, the HDAC2-dependent signaling node we describe here may offer an interesting therapeutic option.

## INTRODUCTION

Transcription factors belonging to the NF-κB family (p65/RelA, p105/p50, p100/p52, RelB, and c-Rel) regulate important physiological processes, such as cell proliferation, development, and immunity. Accordingly, dysregulation of NF-κB proteins can contribute to severe diseases, including cancer [1-3]. The pro-survival role of NF-κB can counteract tumor suppressive pathways, e.g., gene expression signatures induced through p53 [4]. Activation of canonical NF-κB signaling involves ubiquitinylation and phosphorylation events. IκB kinases (IKKs) become activated and phosphorylate inhibitor of NF-κB proteins (IκBs), which are subsequently degraded by the proteasome. The released NF-κB proteins shuttle into the nucleus to activate specific sets of genes [2]. Other activation modes for NF-κB are the non-canonical/alternative and the atypical pathway. The latter is connected to genotoxic stress which activates p53 and NF-κB [4]. The apical stress sensor kinase ATM, IKKε, and IKKγ/NEMO are further players in the control of atypical NF-κB activation [5-8]. Furthermore, increasing evidence shows that p53 directly controls various NF-κB-dependent signaling cascades ultimately determining the balance between cell survival and cell death [4, 9-13].

Lysine/histone deacetylases (KDACs/HDACs) are important epigenetic regulators that catalyze the deacetylation of ε-N-acetylated lysine residues of numerous proteins. The 18 HDACs in humans are sorted into four classes according to phylogenetic derivation, with HDAC2 being a member of class I [14]. On a gene regulatory level, deacetylated histones are traditionally correlated with condensed, transcriptionally inactive chromatin. However, HDAC activity can have positive effects on transcription as well [15]. Furthermore, dynamically regulated non-histone protein acetylation/deacetylation cycles determine gene expression as well as the stability, localization, interaction, and DNA binding affinity of proteins [16]. HDAC1, HDAC2, HDAC4, and SIRT1, are posttranslationally modified by the small ubiquitin-related modifier (SUMO), linking sumoylation to epigenetic regulation [17-20]. Sumoylation is the posttranslational formation of isopeptide bonds between the SUMO C-terminus and the NH2-group of a lysine residue by an enzymatic machinery consisting of a single E1-activating enzyme, the SUMO E2-conjugase UBC9, and several SUMO E3-ligases [21, 22].

P53 functions are antagonized by HDAC2 through its sumoylation-dependent recognition of p53 and a subsequent deacetylation of p53 at K320 [19, 23]. As a result, HDAC2 but not HDAC2^K462R^ enhances the cellular tolerance to DNA damage induced by chemotherapeutics [19]. Less is known about HDAC2 influencing NF-κB-dependent gene expression. Contradictory reports show both, positive and negative influences of HDAC2 on NF-κB [24-27]. Links between p53 and NF-κB and their direct interactions are well established [4, 9, 10, 13, 28, 29]. However, there is still limited information on the mechanisms, posttranslational modifications, and upstream regulators orchestrating the NF-κB/p53 crosstalk [4]. The latter is often found in cells exposed to genotoxic stress which can induce p53 and IKKs triggering NF-κB activation [1]. Furthermore, the kinase RSK1 modulates the p53-dependent activation of NF-κB [11, 12]. It appears possible that histone acetyltransferases (HATs), HDACs, and further epigenetic regulators control NF-κB and its crosstalk with p53.

Here, we demonstrate the importance of HDAC2 for NF-κB-dependent gene expression and analyzed the effect of HDAC2 sumoylation in this process. Indeed, NF-κB-dependent gene expression is increased in the presence of wild-type HDAC2. Sumoylation-deficient HDAC2^K462R^ though fails to propel NF-κB activity. In addition, we reveal that p53 and RSK1 are prerequisites for the positive effect of HDAC2 on NF-κB in colon cancer cells. Furthermore, DNA binding assays show an increased recruitment of p53 to NF-κB consensus binding sequences. Enhanced expression of a subset of NF-κB target genes in cells expressing HDAC2, but not in cells carrying HDAC2^K462R^, suggests a novel, sumoylation-dependent regulatory mechanism for p65. This molecular mechanism appears important for the survival of cancer cells exposed to genotoxic stress.

## RESULTS

### NF-κB-regulated transcription is enhanced by HDAC2 sumoylation

**Figure 1:**
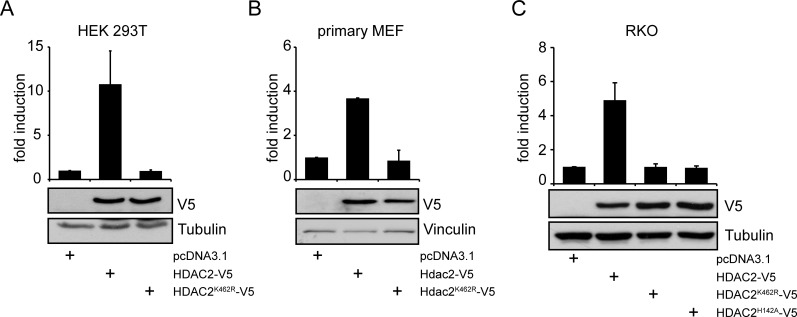
NF-κB-dependent gene expression is regulated in cancer and primary cells by HDAC2 but not by sumoylation deficient HDAC2^K462R^ (A) Luciferase assay in HEK293T cells shows induction of 4xκB-Luciferase (Luc) reporter gene when HDAC2, but not when sumoylation deficient HDAC2^K462R^ is expressed. A representative immunoblot (IB) of n=10 independent experiments for transfection efficiency and loading control is shown. (B) Primary MEFs transfected with murine Hdac2-V5 and Hdac2^K462R^-V5 show induction of a 5xκB-Luc reporter only by wild-type Hdac2. Graph is the mean of n=2 experiments. (C) HDAC2-negative-RKO cells were transfected as in (A), additionally a catalytically not functional HDAC2^H142A^ is coexpressed, n=4. Graphs show mean ± s.e.m. fold induction of luciferase intensity after normalization to activity of cotransfected β-Galactosidase (β-Gal), compared to the pcDNA3.1 transfected control.

Since sumoylation of HDAC2 can control p53 [19], we speculated that HDAC2 sumoylation may also regulate other transcription factors. Activity of NF-κB was previously described to be regulated by HDAC2 and thus seemed to be a worthwhile target to test for effects of HDAC2 sumoylation. To check this hypothesis, we co-transfected a NF-κB luciferase reporter with expression vectors encoding wild-type HDAC2 or HDAC2^K462R^ [19]. Expression of HDAC2 significantly activated the NF-κB-dependent reporter while HDAC2^K462R^ did not induce NF-κB activity under identical conditions (Figure 1A). Likewise, HDAC2, but not HDAC2^K462R^ overexpression caused induction of NF-κB luciferase reporters with different numbers of κB consensus DNA binding sites (Supplementary Figure S1A).

Next, we analyzed whether HDAC2 can also activate NF-κB-dependent gene expression in other cell types including non-transformed cells. We conducted experiments with primary murine embryonic fibroblasts (MEFs). Murine HDAC2 shows conservation of the sumoylation site but differs from the human sequence at two amino acid positions. Therefore, the murine *Hdac2* cDNA was cloned into the same expression vector system we used to express human HDAC2. Like in the transformed cells, murine HDAC2, but not HDAC2^K462R^, activated the NF-κB-dependent reporter (Figure 1B). Additionally, transient expression of HDAC2 in the HDAC2-negative RKO cell line [30] induced NF-κB activity, while the sumoylation-deficient mutant HDAC2^K462R^ did not (Figure 1C). We then tested whether the control of NF-κB via HDAC2 relies on the deacetylase activity of HDAC2. We analyzed how the catalytically inactive HDAC2^H142A^ [19] affects NF-κB-dependent reporter gene expression. Like HDAC2^K462R^, HDAC2^H142A^ failed to activate the NF-κB reporter in RKO (Figure 1C) and HEK293T cells (Supplementary Figure S1B).

Mutation of K462 may also abrogate posttranslational modifications other than sumoylation. Thus, we also tested HDAC2 mutants of the surrounding ΨKxE sumoylation-consensus motif (Supplementary Figure S1C). The mutants HDAC2^V461A^ and HDAC2^E464A^, which cannot become sumoylated [19], are not able to induce the NF-κB-dependent reporter. In contrast, mutation of the sumoylation-irrelevant x position (HDAC2^E463A^) retains the ability of HDAC2 to activate NF-κB-dependent luciferase expression (Supplementary Figure S1C,1D).

### HDAC2 regulates unstimulated, endogenous NF-κB-dependent gene expression and apoptosis following genotoxic stress

**Figure 2:**
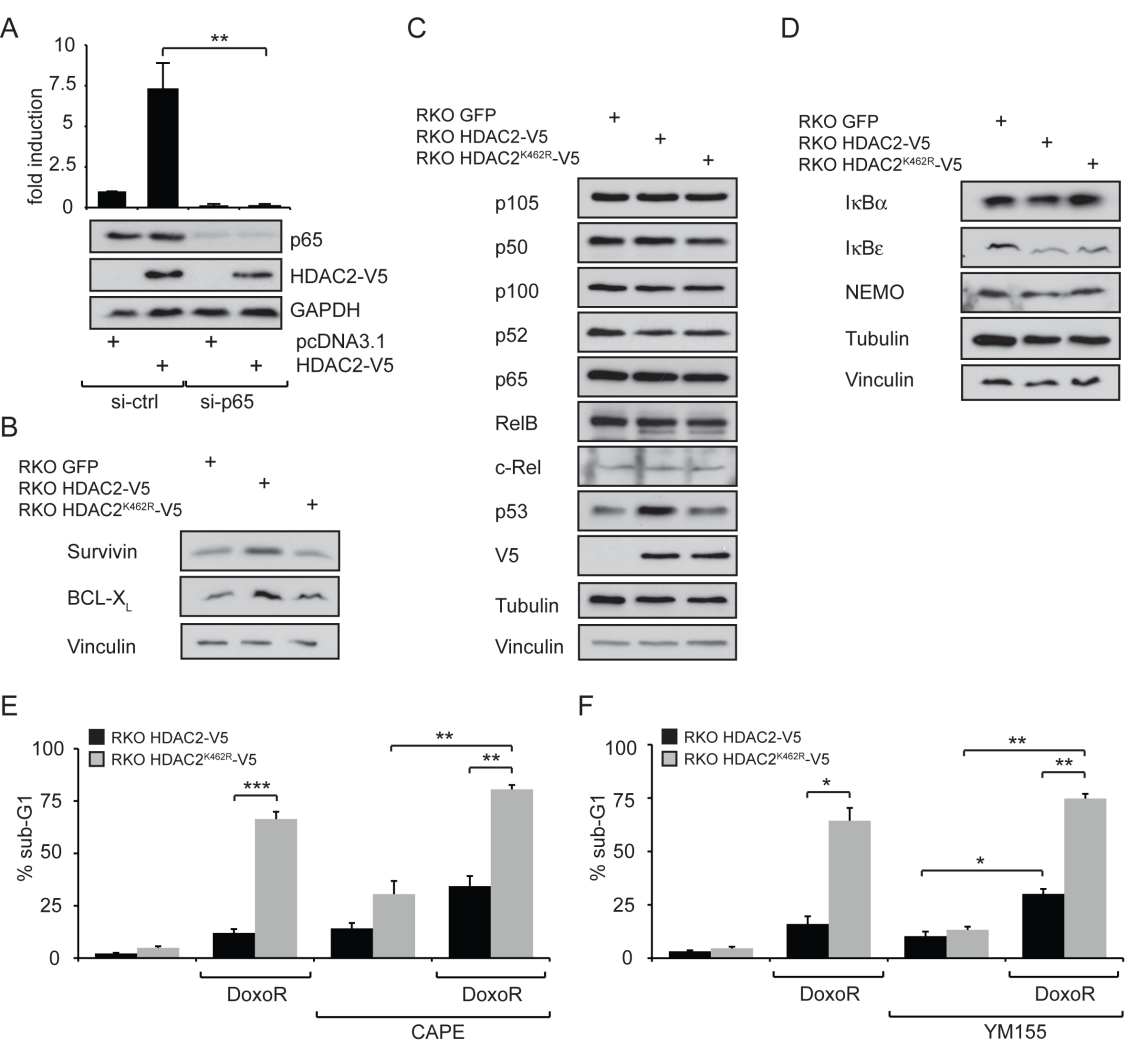
Expression levels of NF-κB target genes and influence on genotoxic stress tolerance in a model cell system comparing wild-type HDAC2 and non-sumoylatable HDAC2^K462R^ (A) Knockdown of p65 with siRNA confirms that HDAC2 acts directly via NF-κB on the reporter. Here: n=4; **=p<0.01. (B) Expression levels of NF-κB target genes survivin and Bcl-X_L_ in stably transfected RKO GFP, RKO HDAC2-V5 and RKO HDAC2^K462R^-V5. Increased activity of NF-κB in the presence of HDAC2 results in slightly enhanced expression of the NF-κB targets survivin and Bcl-X_L_ in cells carrying HDAC2 compared to HDAC2^K462R^. (C) Expression of the indicated NF-κB proteins or (D) the indicated NF-κB regulatory proteins is not grossly different between RKO GFP, RKO HDAC2-V5 and RKO HDAC2^K462R^-V5 colon cancer cells. (E) Influence on genotoxic stress tolerance in a model cell system comparing wild-type HDAC2 and non-sumoylatable HDAC2^K462R^. Apoptosis induction in RKO HDAC2-V5 and HDAC2^K462R^-V5 cells after 24 h treatment with 1 μM Doxorubicin (DoxoR) in combination with 25 μM NF-κB inhibitor caffeic acid phenethyl ester (CAPE). CAPE was added 30 min prior to DoxoR. (F) As in (E), but cells were coincubated with 1 μM DoxoR in combination with 100 nM of the survivin suppressant YM155 as indicated. Bar diagrams show mean ± sem of % sub-G1 fraction of n=5 (E) or n=3 (F) experiments, **=p<0.01; ***=p<0.001.

The HDAC2-mediated induction of the NF-κB luciferase reporter could be a direct effect on the NF-κB pathway or a general regulation of the transcriptional machinery. As shown in Figure 2A, siRNA against p65 completely abolished the HDAC2-dependent activation of the NF-κB luciferase reporter in RKO cells, illustrating that HDAC2 induction of this reporter functions through p65. Similar results were obtained using siRNA against RelB (Supplementary Figure S2A). Treatment with TNFα induced the luciferase reporter expression regardless if cells were transfected with HDAC2 or HDAC2^K462R^ (Supplementary Figure S2B). There was a small but insignificant trend that the NF-κB luciferase reporter is activated stronger by TNFα in the presence of HDAC2 compared to HDAC2^K462R^ or control (Supplementary Figure S2B). Thus, the reporter system remains cytokine-inducible irrespective of HDAC2 sumoylation.

Our previous results demonstrated that cells stably transfected with HDAC2 and HDAC2^K462R^ (RKO HDAC2-V5 and RKO HDAC2^K462R^-V5, respectively) showed differences in their resistance toward genotoxic stress [19]. Accordingly, we asked whether pro-survival NF-κB target genes could be responsible for the protection of cells with wild-type HDAC2. We found a slight upregulation of the anti-apoptotic proteins BCL-X_L_ and survivin in RKO HDAC2-V5 compared to RKO HDAC2^K462R^-V5 cells (Figure 2B). However, the levels of numerous other classical NF-κB target genes were not altered in their expression (data not shown). The levels of NF-κB family members (Figure 2C) and of proteins controlling the NF-κB pathway (Figure 2D) were not different in cells stably reconstructed with HDAC2 or HDAC2^K462R^. These results disfavor altered levels of these factors as an explanation for the HDAC2-dependent NF-κB signaling activities we reveal here.

Next, we tested whether NF-κB-dependent survival signaling is enhanced by HDAC2 but not by HDAC2^K462R^ in RKO cells. The anthracycline doxorubicin is a genotoxin by virtue of its abilities to intercalate into DNA and to block topoisomerase II [31, 32]. It is far more toxic to cells expressing sumoylation-deficient HDAC2 [19] and we wanted to find out whether this might be linked to NF-κB activity in HDAC2-positive RKO cells. Therefore, we added the NF-κB inhibitor caffeic acid phenethyl ester (CAPE) to RKO HDAC2-V5 and RKO HDAC2^K462R^-V5 cells; CAPE blocks the DNA binding of NF-κB proteins [9]. We noted that CAPE enhanced doxorubicin-induced apoptosis rates in RKO HDAC2-V5 and in RKO HDAC2^K462R^-V5 cells (Figure 2E). Moreover, RKO HDAC2-V5 cells are sensitive for CAPE even without additional doxorubicin treatment (Figure 2E), illustrating that these cells rely on NF-κB-dependent survival signaling. These data suggest that interfering with NF-κB attenuates survival signaling protecting RKO HDAC2-V5 cells.

Figure 2B shows enhanced survivin expression in RKO HDAC2-V5 cells and we hence investigated whether inhibition of survivin with YM155 is able to sensitize RKO HDAC2-V5 cells to genotoxic stress caused by doxorubicin. YM155 antagonizes survivin, some other NF-κB-regulated signaling molecules, and further pathways [33]. We found that YM155 promotes apoptosis in such cells treated with doxorubicin (Figure 2F). From these data we conclude that the attenuation of NF-κB and survivin can increase the sensitivity of RKO cells harboring wild-type HDAC2 to an anthracycline causing DNA damage.

### Induction of NF-κB activity by HDAC2 depends on p53

**Figure 3:**
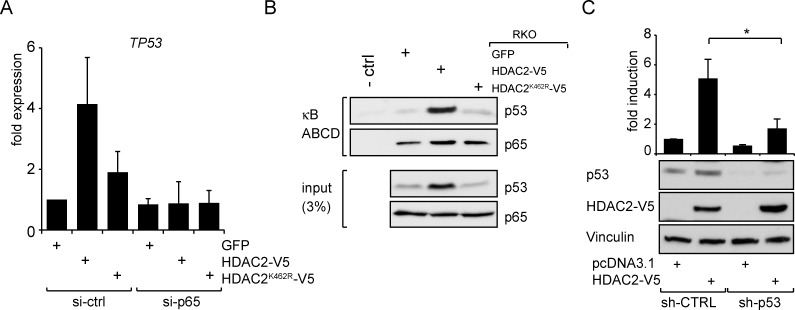
HDAC2 influences NF-κB-dependent TP53 expression and p53 crosstalks with NF-κB to regulate NF-κB transcriptional activities (A) qPCR of TP53 in RKO cells stably transfected as indicated and additional knockdown of p65. Diminishing p65 abrogates enhanced *TP53* expression. (B) ABCD assay showing p53 binding to NF-κB complexes using RKO cells stably transfected with HDAC2-V5, HDAC2^K462R^-V5 or GFP for control. The amount of p53 recruited to κB consensus sites is dependent on the HDAC2 status. More p53 is recruited to NF-κB when wild-type HDAC2 is present. (C) p53 knockdown attenuates the induction of NF-κB reporter gene by HDAC2; n=5.

The tumor suppressor p53 has an important impact on NF-κB-dependent gene expression by interaction with NF-κB [9] and via indirect mechanisms [4, 10]. We therefore tested for a functional contribution of p53 toward the HDAC2-dependent control of NF-κB. Interestingly, p53 is an NF-κB target [34, 35] and this should result in higher *p53* mRNA levels in cells with enhanced NF-κB activity. Indeed, *p53* mRNA levels were higher in RKO cells expressing wild-type HDAC2 (Figure 3A). Consistently, protein levels of p53 are higher in these cells (see Brandl *et al*. 2012 and Figure 2C). Furthermore, after diminishing NF-κB p65 by RNAi the expression of *Tp53* in RKO cells became reduced (Figure 3A). These data point out that *TP53* is a p65/HDAC2 target gene in our cell system. We then investigated whether NF-κB-dependent transcription induced by HDAC2 relies on the presence of p53 in NF-κB transcriptional complexes. As shown in Figure 3B, p53 is recruited to NF-κB protein complexes which were precipitated in an ABCD assay by oligonucleotides carrying κB consensus binding sequences. The interaction is stronger in lysates from RKO cells expressing HDAC2, but not in cells expressing HDAC2^K462R^ or GFP for control (Figure 3B).

Interestingly, the results depicted in Figure 3C further demonstrate that p53 is necessary for the activation of NF-κB through HDAC2. The knockdown of p53 with shRNA diminishes the induction of NF-κB reporter gene expression caused by HDAC2 (Figure 3C). Many p53 functions are dependent on its acetylation and HDAC2 can specifically reduce the acetylation of p53 at K320 [19]. We considered that a p53 molecule mimicking p53 acetylation at K320 (pseudo-acetylated p53^K320Q^) may abrogate positive effects of p53 on NF-κB. To test this, we used the NF-κB-dependent luciferase assay and the reported possibility to activate it through p53 overexpression [11, 12]. However, reconstitution of p53-negative HCT 116 colon cancer cells with wild-type p53 or p53^K320Q^ similarly activated NF-κB reporter gene expression (Supplementary Figure S3A). This may exclude that p53^K320^ acetylation has an impact on NF-κB expression.

### Modulation of the nuclear localization of p65 by HDAC2 and by genotoxic stress

**Figure 4:**
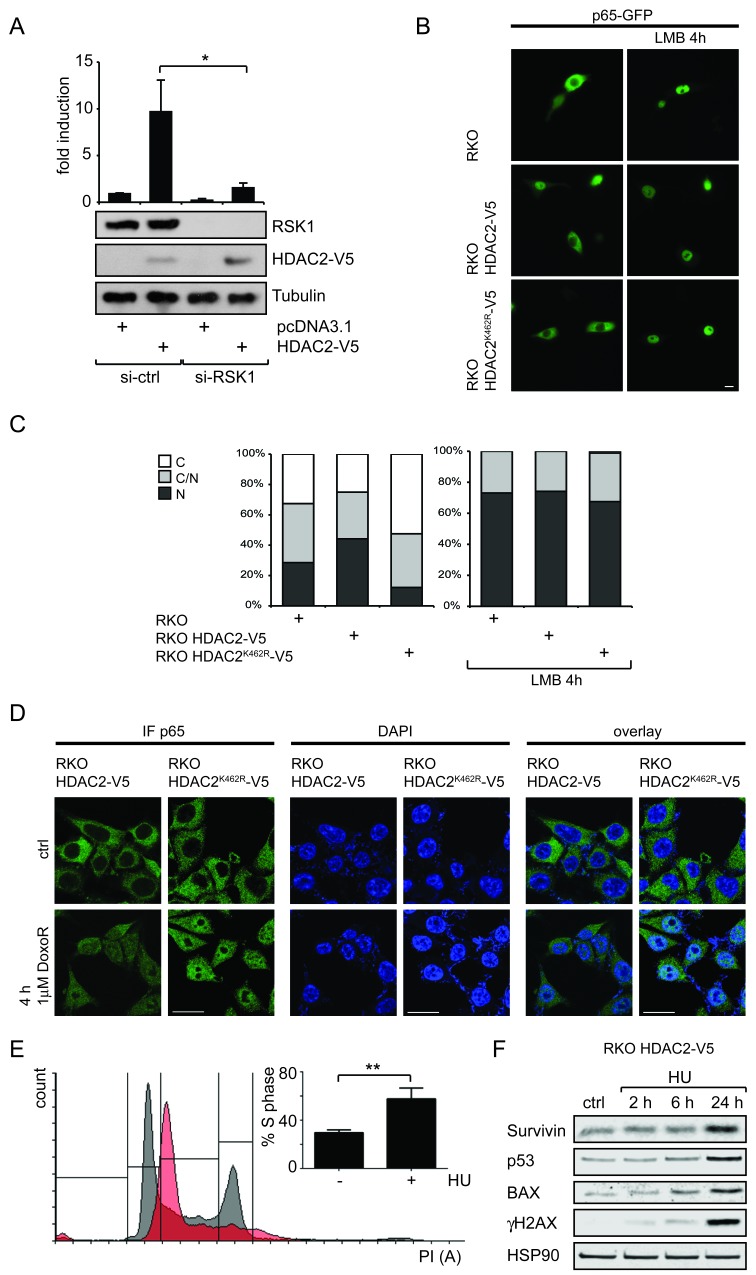
Connection of RSK1 activity and subcellular localization of p65 toward HDAC2 induction of NF-κB (A) Knockdown of RSK1 diminishes HDAC2-caused NF-κB reporter gene induction; n=4. (B) Representative image of RKO, RKO HDAC2-V5 and RKO HDAC2^K462R^-V5 cells transfected with p65-GFP, showing enhanced nuclear localization of p65 in RKO HDAC2-V5 cells. Inhibition of nuclear export by Leptomycin B (LMB) leads to nuclear retention of p65-GFP regardless of HDAC2 status. (C) Quantification of (D) counting at least 100 cells in two independent experiments. C and N depict predominantly (> 80%) cytosolic or nuclear localization of p65-GFP, respectively, C/N = intermediate distribution of p65-GFP. (D) RKO HDAC2-V5 and RKO HDAC2^K462R^-V5 cells were treated with 1μM doxorubicin (DoxoR) for 4 h or left untreated for control. Fixed cells were stained with antibodies against p65 and visualized with Cy2 or Cy3 labeled secondary antibody. DNA was stained by mounting medium containing DAPI. Brightness levels for p65 were adjusted for RKO HDAC2-V5 ctrl (45), RKO HDAC2^K462R^-V5 ctrl (35) and DoxoR (35, all from original 50). Brightness for DAPI was adjusted for RKO HDAC2^K462R^-V5 cells DoxoR (to 45 from original 50). Scale bar = 20 μm. (E) RKO HDAC2-V5 cells were treated with 1.5 mM Hydroxyurea (HU) for 24 h and cell cycle distribution was analyzed by PI staining. The bar diagram shows the amount of cells in S-Phase of n=5 experiments; ± sem, ** = p<0,01. A histogram for one representative experiment shows cell cycle profile of control cells in black and of HU treated cells in red. (F) RKO HDAC2-V5 cells were treated with 1.5 mM HU for 2, 6 and 24 h or left untreated for control. Levels of the indicated proteins were analyzed by Western blot.

To gain further insights into how HDAC2 controls NF-κB-dependent transcription, we investigated whether HDAC2 alters NF-κB activity through the kinase RSK1, which is necessary for the p53-dependent gene expression controlled by NF-κB [12]. Indeed, the knockdown of RSK1 led to a strong decrease of NF-κB reporter gene activity and blunted the stimulating effect of HDAC2 on NF-κB (Figure 4A). From these observations we conclude that RSK1 is required for the activation of NF-κB by HDAC2.

Since p53 is nuclear in RKO cells (Supplementary Figure S3B), the interaction between p65 and p53 in HDAC2 reconstituted RKO cells may affect the subcellular localization of p65. Furthermore, RSK1 is also able to influence the subcellular localization of NF-κB p65 [12]. We determined the intracellular distribution of p65 by fluorescence microscopy in HDAC2-negative RKO cells, RKO HDAC2-V5 and RKO HDAC2^K462R^-V5 cells. We additionally transfected the cells with p65-GFP, to monitor p65 via the GFP signal and to increase the pool of p65. In the presence of HDAC2, a significant portion of p65-GFP is shifted to the nucleus, while RKO and RKO HDAC2^K462R^-V5 cells show a more cytosolic distribution of p65-GFP (Figure 4B). In agreement with these data, it has been found that HDAC2 levels are often elevated in pancreatic cancers and that tumors showing high HDAC2 expression also have increased nuclear p65 levels [36, 37]. Treatment with leptomycin B (LMB) was used to exclude general defects in the nuclear export machinery in RKO cells. As expected, LMB treatment traps p65-GFP to the nucleus regardless of the HDAC2 status of the cells (Figure 4C). Thus, an altered p53 interaction with p65 in cells expressing HDAC2 could explain the increased nuclear localization of p65.

Further, we tested the subcellular distribution of endogenous p65 in RKO HDAC2-V5 and RKO HDAC2^K462R^-V5 cells exposed to genotoxic stress induced by doxorubicin. We had previously reported that doxorubicin decreased the conjugation of SUMO1 to HDAC2 [19] and we therefore asked if p65 translocated to the nucleus equally in the doxorubicin-treated RKO HDAC2 wild-type/HDAC2^K462R^ cell pair. Indeed, this drug triggered the accumulation of p65 in in both cell types (Figure 4D) suggesting that this process is not hampered by HDAC2.

Doxorubicin only induces NF-κB target genes in a p53 null background [38], but wild-type p53 is present in RKO cells [19]. Therefore, we used hydroxyurea (HU). This drug causes replicative stress by ribonucleotide reductase inhibition and subsequent dNTP depletion and DNA damage via the breakdown of replication forks [4]. The analysis of cells exposed to HU allowed us to test if the suppression of p53 target genes by HDAC2 [19] and the effect of HDAC2 on NF-κB, which we show here, persist upon activation of the replicative checkpoint with HU. We could successfully stall RKO HDAC2-V5 cells in S phase (Figure 4E). HU blocks replication fork progression and/or induces DNA damage as seen by an accumulation of γH2AX, and an induction of p53, which is accompanied by an induction of its target gene BAX. However, HU-induced p53 does not trigger a repression of survivin (Figure 4F). These results suggest that replicative stress can relieve the suppression of p53 activity by wild-type HDAC2, but that this accumulation of p53 also leads to an accumulation of survivin in such cells.

## DISCUSSION

NF-κB-dependent gene expression can be both, positively and negatively affected by HDAC2 [24-26]. Here we show that HDAC2 enhances NF-κB-dependent gene expression in tumor-derived cell lines and in primary cells (Figure 1A-C). HDAC2 has a typical ΨKxE sumoylation consensus motif (VK^462^EE in HDAC2) in its unstructured C-terminal domain. Mutating K462, V461, or E464 abrogates the posttranslational modification of HDAC2 with SUMO1 [19]. Interestingly, mutants of HDAC2 which are sumoylation-deficient do not show an influence toward NF-κB activity and neither does a catalytically inactive HDAC2. In order to investigate the influence of HDAC2 on the expression of endogenous NF-κB target genes, we used stably transfected RKO cell lines. The expression of NF-κB family members and proteins controlling the NF-κB activation pathway is not altered in the cellular models investigated (Figure 2C, 2D), excluding altered regulation of NF-κB at this level. In RKO cells stably expressing wild-type HDAC2, numerous classical NF-κB target genes including IκBα are also not altered in their expression levels. In agreement with these data, IκBα was previously reported to be regulated independently of HDAC2 [39]. However, stimulation of RKO cells with the cytokine TNFα verified that NF-κB signaling evoked by classical IKK/IκB activation is intact in RKO cells expressing HDAC2 or HDAC2^K462R^ (Supplementary Figure S2B). Thus, there is no general defect in NF-κB in RKO cells expressing the HDAC2 sumoylation mutant.

Strikingly, the transcription of the gene coding for p53 was enhanced when HDAC2 was present and this could be abrogated by a p65 knockdown (Figure 3A). *T*P*53* carries an evolutionary conserved consensus κB-binding site [34, 40]. Accordingly, the expression of TP53 is diminished when NF-κB is blocked [34] and recent data show that a knockdown of p65 decreases p53 protein levels [35]. Many mechanisms including post-transcriptional events regulate p53 protein levels. Still, enhanced NF-κB activity could in part explain our previous observation of enhanced - yet functionally inactive - p53 in cells with HDAC2 [19]. Levels of the anti-apoptotic NF-κB target genes BCL-X_L_ and survivin were slightly elevated in cells carrying HDAC2 compared to cells expressing sumoylation-deficient HDAC2^K462R^ (Figure 2B). Again, other factors could tie into the regulation of both genes. Expression of survivin can be downregulated by active p53 and enhanced survivin levels by HDAC2 might be the result of both, elevated NF-κB activity and p53 inactivation [19]. Consistent with the higher levels of anti-apoptotic protein expression, blocking NF-κB activity with CAPE or YM155 led to an increased rate of apoptosis (Figures 2E, 2F). These findings are coherent with previous results, which were collected in cancer cells treated with doxorubicin and inhibitors of NF-κB signaling initiated in the cytosol (reviewed in [38]). The higher sensitivity of colon cancer cells lacking wild-type HDAC2 is also in agreement with the observation that a knockdown of HDAC2 in pancreatic ductal adenocarcinoma (PDAC) has no effect on cell viability but sensitizes PDAC cell lines to etoposide-induced apoptosis [37]. Our novel data show that sumoylation of HDAC2 provides a survival benefit compared to non-sumoylatable HDAC2^K462R^ and that NF-κB contributes to this phenotype. Replicative stress caused by dNTP depletion blocks replication fork progression. This also liberates p53′s activating functions on transcription, but not its repressive effects on survivin (Figure 4F). Here, a p53/NF-κB crosstalk [9] seems to operates and to exert a dominant effect on survivin expression.

Sumoylation often alters the interactome of the modified proteins [41]. However, we did not observe that interaction of p65 with HDAC2 or that the acetylation of p65^K310^ was dependent on the sumoylation of HDAC2 (data not shown). These findings are consistent with the literature that speaks against the deacetylation of p65 by HDAC2; HDAC1 and HDAC3 are though commonly found to counteract p65 acetylation [42-44]. Moreover, HDAC2 and HDAC3 interact with acetylated p65, but only HDAC3 is able to deacetylate p65 [43]. In murine neuronal models, p65 acetylation and downstream effects were not affected by either HDAC1 or HDAC2 alone; only when both were disrupted the acetylation of p65 was augmented [44]. Other studies equally suggest that HDAC2 cannot directly bind p65 [25], and the interaction between HDAC2 and NF-κB may require very specific conditions *in vivo*. It is conceivable that inconsistent results reported for the interaction between HDAC2 and NF-κB [24-27], rely on differences in the cellular p53 status, HDAC1/HDAC2 heterodimerization, or altered RSK1 activity. However, deacetylation of p53 or p65 unlikely explains the stimulating effect of HDAC2 on NF-κB in our model system.

Crosstalk between p53 and NF-κB family members contributes to oncogenesis, metastasis, and immune functions [9, 11, 28, 29]. A recent study shows that NF-κB transcriptional activity in breast cancer cells depends on their p53 mutational status [38]. Only cells with mutated p53 showed upregulation of NF-κB target genes after treatment with the chemotherapeutic doxorubicin. However, the set of upregulated genes was varying between different tumor cells [38]. Our results extend the findings by Dalmases and colleagues by showing that sumoylated HDAC2, which also specifically causes a functional deficiency in p53, promotes NF-κB-dependent gene expression in colon cancer cells. Moreover, a prolonged NF-κB activity is also observed when p53 is mutated in colon cancers [29] and an NF-κB-dependent inflammatory response critically contributes to tumorigenesis [45]. Remarkably, previous data showed that inhibiting the IKK complex blocks the doxorubicin-induced nuclear accumulation of p65 (reviewed in [38]). We demonstrate that the control of NF-κB p65 by doxorubicin and by the cytokine TNFα (Figures 4D and S2B) is independent of HDAC2 sumoylation and dominant over HDAC2-dependent effects. The control of NF-κB by HDAC2 requires p53 and RSK1 and rather seems to be a nuclear event that is likely independent of IKK.

**Figure 5:**
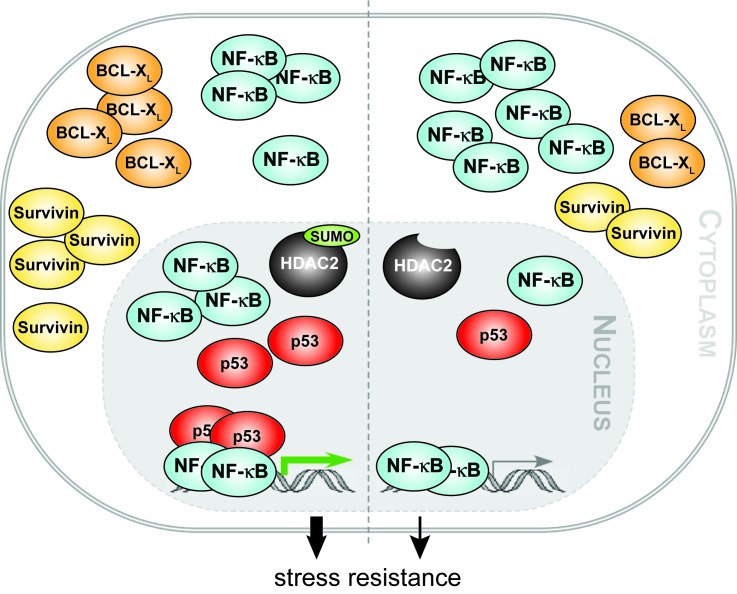
HDAC2 sumoylation integrates NF-κB signaling Our work reveals that the epigenetic regulator histone deacetylase 2 (HDAC2) can activate NF-κB-dependent gene expression in human cancer cells and in murine embryonic fibroblasts. We show that the posttranslational modification of HDAC2 with the small ubiquitin-related modifier (SUMO) integrates NF-κB p65 and the tumor suppressor p53. In the absence of HDAC2 sumoylation the activation of NF-κB by HDAC2 is strongly diminished. Our data further suggest that enhanced recruitment of p53 to NF-κB p65 bound to DNA and an increased nuclear accumulation of p65 are causal for an augmented NF-κB-dependent anti-apoptotic gene expression in cells expressing wild-type HDAC2. Additionally, we demonstrate that HDAC2, but not its sumoylation-deficient variant, increases the resistance of colon cancer cells toward genotoxic stress. These findings suggest that the HDAC2-NF-κB signaling node we report in our manuscript contributes to tumorigenesis and chemoresistance.

RSK1 has been described to be an important factor in coordinating the p53 crosstalk in situations when NF-κB is not activated by classical stimuli [12]. Accordingly, knockdown of either p53 or RSK1 in our setting abrogates the effect of HDAC2 towards NF-κB (Figures 3C and 4A). Additionally the presence of HDAC2 results in enhanced recruitment of p53 to NF-κB transcriptional complexes (Figure 3B). This cross-signaling between p53 and NF-κB proteins gives further insight in molecular mechanisms regulating tumorigenesis and might be a point of action for pharmacological intervention [9, 28, 29, 46]. In sum, our data show that HDAC2 sumoylation is important for NF-κB-dependent gene expression and for the resistance of cell against stress (Figure 5). Hence, monitoring the expression of HDAC2 and its sumoylation status could be of prognostic value and HDAC2 appear as a promising target for therapeutic intervention strategies.

## MATERIAL and METHODS

### Cell culture, chemicals, and generation of mouse embryonic fibroblasts

HEK 293T, HCT 116 p53^−/−^ cells [47], RKO cells lacking HDAC2 [48], RKO cell lines stably transfected with HDAC2 (wild type or HDAC^K462R^) and GFP [19], and mouse embryonic fibroblast (MEF) cell lines were cultivated at 37°C and 5% CO_2_ in Dulbecco's modified Eagle's medium (DMEM) containing 2% L-glutamine (PAA; Cölbe, Germany) + 10% FCS (Sigma-Aldrich). Primary MEFs were isolated from 13.5 dpc wild type (wt) and *Hdac2^−/−^* embryos as described [49]. Primary MEFs were frozen at passage 1 and used for experiments up to passage 6.

D-Luciferin potassium salt was purchased from Biomol (Hamburg, Germany), doxorubicin (DoxoR) and caffeic acid phenethyl ester (CAPE) from Enzo (Lörrach, Germany), TNFα from ReliaTech (Wolfenbüttel, Germany), N-ethylmaleimide (NEM), Leptomycin B, and YM155 from Selleckchem (Munich, Germany). All other chemicals were purchased from Carl Roth (Karlsruhe, Germany).

### Transfections, luciferase assays, and plasmids

Plasmids encoding wild-type human HDAC2, mutants derived thereof, and pSV40-β-GAL4 [19], pSUPER-p53 and -ctrl [50], 3xκB-Luc [51] were described previously. 4xκB- and 5xκB-Luc were generated from 3xκB-Luc and kindly provided by J. Schmid (Vienna, Austria). A construct expressing V5-tagged murine Hdac2 was generated by TOPO®-cloning in pcDNA3.1 vector backbone (Life Technologies). Hdac2^K462R^-V5 was derived thereof by QuickChange Site-Directed Mutagenesis (Agilent, Frankfurt/Main, Germany). To generate p65-GFP, the p65 coding sequence was amplified from cDNA obtained from a human head and neck tumor as described [52], inserted into the pF25 expression vector using *NheI* restriction sites and verified by sequencing as described [53, 54]. siRNA pools targeting p65 and HDAC2 were purchased from Santa Cruz, RSK1 (RPS6KA1) from Thermo and RelB siRNA was published previously [55].

HEK 293T, HCT- 116 p53^−/−^ and all RKO cells were transfected with Lipofectamine^TM^ 2000 (Life Technologies, Darmstadt, Germany) according to the manufacturer's protocol. Transfections for luciferase assays were performed in 24-well plates in triplicate. 0.1 μg pSV40-β-GAL, 0.3 μg luciferase reporter construct (3x/4x/5x-κB-Luc) and 0.4 μg HDAC2 (HDAC2-V5, HDAC2^K462R^-V5, HDAC2^H142A^-V5) were used per well. In p53 knockdown experiments, 0.1 μg sh-p53 (pSUPER-p53) and 0.3 μg HDAC2 or empty vector were used. In experiments with siRNA knockdown 20 pmol siRNA were cotransfected with a total of 0.4 μg plasmids (0.05 μg pSV40-β-GAL, 0.05 μg κB-Luc, 0.3 μg HDAC2). Primary MEFs were transfected with Attractene (Qiagen, Cologne, Germany) according to the manufacturer's protocol (0.2 μg pSV40-β-GAL, 0.3 μg 5x-κB-Luc, and 0.5 μg HDAC2 plasmids). For controls, empty vector (pcDNA3.1), sh-ctrl (pSUPER-sh-scrambled) and siRNA-ctrl were used. Cells were lysed in luciferase harvest buffer (37.5 mM Tris-HCl pH 8.0, 12.5 mM 2-(N-Morpholino)ethanesulfonic acid (MES), 10% glycerol, 0.1% Triton-X 100, 1 mM DTT, PIC) 48 h after transfection. The luciferase reporter activity was normalized to the activity of the cotransfected β-galactosidase reporter.

### ABCD assays, Western blotting, antibodies

For ABCD assays cells were seeded in 10 cm dishes, harvested after 24 h and lysed in NETN buffer (150 mM NaCl, 20 mM Tris/HCl pH 7.4, 0.5% NP-40, 10% glycerol, 1 mM EDTA, 10 mM NEM, 1 mM PMSF, protease inhibitor cocktail [PIC; end concentrations 4 μg/ml antipain, 20 units/ml aprotinin, 0.2 mg/ml benzamidine, 2 μg/ml leupeptine]). Oligonucleotides with a NF-κB consensus site (sense oligo: bio-5′-GGAATTTCCGGGAATTTCCGGGAATTTCCGGGAATTTCCC) [9] were used to pull down protein complexes and then analyzed by Western Blot as described [19, 56]. 300 μl lysate was incubated together with biotinylated oligonucleotides for 30 min on ice. For pull down of biotinylated oligonucleotides together with bound proteins streptavidin beads were added to the lysates and put on a rotating wheel for 1 h. As negative control, biotinylated oligonucleotides with a scrambled binding site were incubated with a combination of all lysates used. Bound proteins were eluted from the beads with 2x Laemmli buffer and analyzed by western blotting.

SDS-PAGE and immunoblot were performed as described [57]. Equal loading was controlled with housekeeping proteins. When reprobes were not feasible, same protein amounts from cell lysate were loaded on separate gels to perform parallel IBs. Antibodies used are: mouse α-BAX (2D2), rabbit α-Bcl2, rabbit α-c-Rel (N-466), rabbit α-HDAC2 (H-54), mouse α-HSP90 (F-8), mouse α-IκBα (H-4), rabbit α-IκBβ (C-20), rabbit α-IκBε, rabbit α-MCL1 (S-19), rabbit α-mSIN3A (K-20), rabbit α-NEMO (FL-419), rabbit α-p50 (H-119), mouse α-p53 (DO-1), mouse α-p65 (F-6), rabbit α-p65 (C-20), rabbit α-RelB (H-200), rabbit α-RSK1 (C-21) (all Santa Cruz); mouse α-Bcl-x (BD Pharmingen), mouse α-γH2AX (pS139) (Millipore), mouse α-p52 (Upstate), rabbit α-survivin (Novus), mouse α-alpha-Tubulin (Sigma-Aldrich), mouse α-V5 (Life Technologies). Secondary antibodies goat α-rabbit and goat α-mouse are from Pierce/Thermo.

### qPCR

RNA was isolated with RNeasy Kit (Qiagen) and transcribed into cDNA with RevertAid cDNA Synthesis Kit (Thermo). RT-qPCR with primers for *TP53* (fwd: GCCCCCAGGGAGCACTA; rev: GGGAGAGGAGCTGGTGTTG) [58] and housekeeping genes *RPL13A* and *HMBS* [19] was carried out as described [57].

### Fluorescence microscopy and flow cytometry

RKO, RKO HDAC2-V5 and RKO HDAC2^K462R^-V5 cells were transiently transfected with p65-GFP as described above. After 24 h, protein localization was analyzed in live cells with an AxioVert100 fluorescent microscope (Carl Zeiss, Jena, Germany). Alternatively, cells were incubated with 10 nM leptomycin B (LMB) 4 h before analysis. To determine the average intracellular localization of p65-GFP, at least 100 fluorescent cells were examined in two independent experiments. The number of cells exhibiting cytoplasmic (C; cytoplasmic signal >80% of the total cellular signal), cytoplasmic and nuclear (C/N) or nuclear (N; nuclear signal >80% of the total cellular signal) fluorescence was visually inspected and counted. For staining of endogenous p53 mouse α-p53 (DO-1) was used as described [19]. Images were captured using a digital AxioCam CCD camera with the AxioVision Software (Carl Zeiss).

For visualization of endogenous p65 in RKO HDAC2-V5 and RKO HDAC2^K462R^-V5, cells were grown on cover slides and treated as indicated. After treatment, cells were fixed with methanol, incubated with primary mouse α-p65 (diluted 1:50) or rabbit α-p65 (1:100) antibodies and secondary α-mouse antibodies coupled to Cy2 (1:400) or α-rabbit antibodies coupled to Cy3 (1:400), respectively (Jackson Immunoresearch, West Grove, PA, USA). DNA staining was carried out by Prolong Gold Antifade mounting medium with DAPI (Invitrogen, Carlsbad, USA). Images were acquired with a Zeiss LSM 710 laser scanning confocal microscope (Carl Zeiss, Jena, Germany). Brightness levels were adjusted as indicated.

Flow cytometry analysis to determine cell cycle distribution by propidium iodide staining was performed as described [19].

### Statistical analysis

Graphs show means of independent experiments ± standard errors (s.e.m.) if not indicated otherwise in the figure legends. Two-tailed unpaired Student's t-test was used to analyze differences between two groups of samples. Significance was set at p<0.05 if not indicated otherwise in the figure legend.

## SUPPLEMENTARY MATERIAL FIGURES


