# Supplementary material for: Sumoylation of HDAC2 promotes NF-κB-dependent gene expression
Source: Oncotarget. 2015 Jan 21;6(9):7123–35. doi: 10.18632/oncotarget.3344 (PMC4466673; doi:10.18632/oncotarget.3344)
Supplement: Supplementary file 1 [file oncotarget-06-7123-s001.pdf]

Sumoylation of HDAC2 promotes NF-κB-dependent gene expression

Supplementary Material

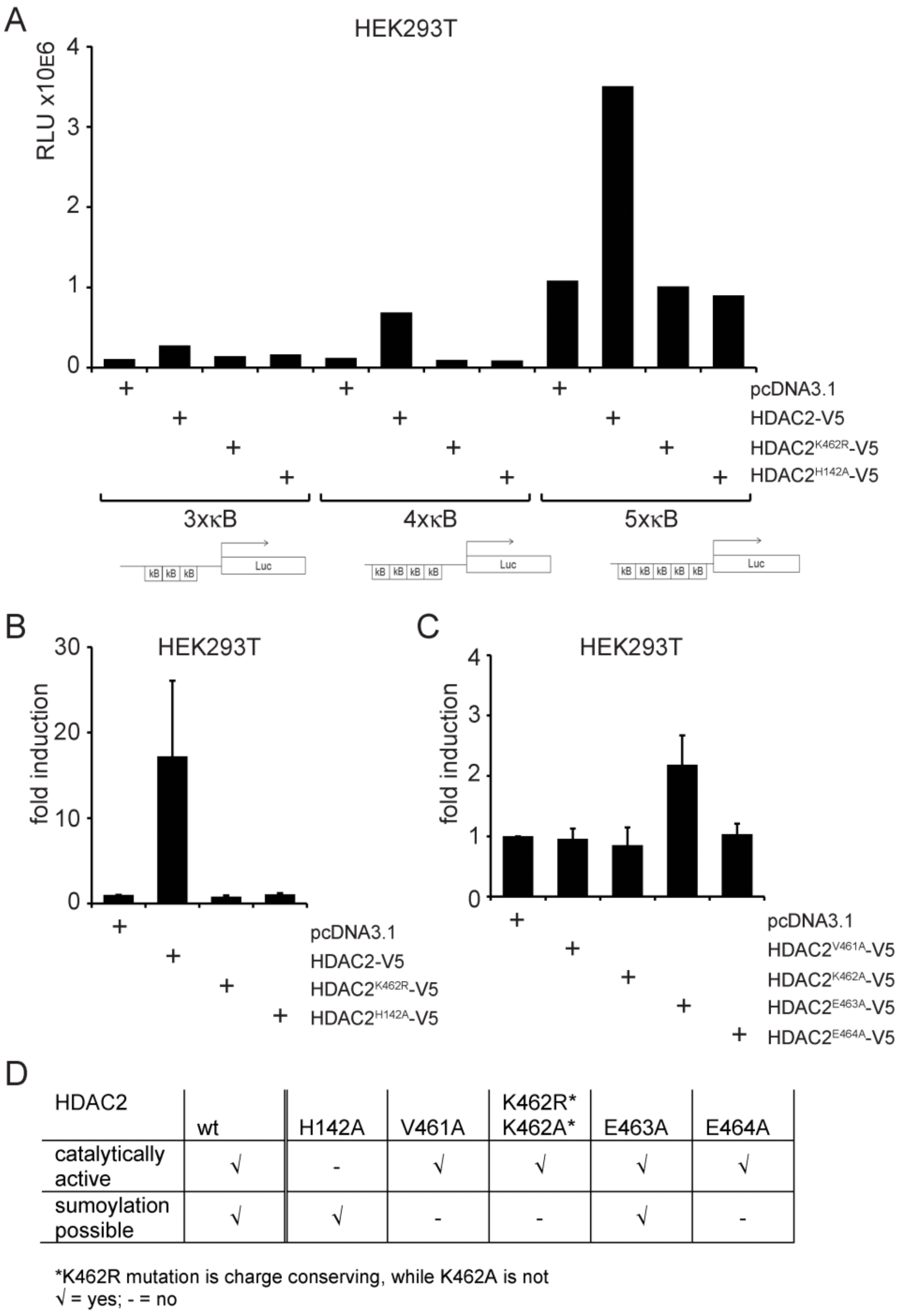

Supplementary Figure S1: (**A**) Representative diagram of κB-Luc reporter gene assay in HEK293T cells using reporter constructs with increasing number of κB consensus sites (3/4/5) with cotransfection of HDAC2 variants or empty vector as in Figure 1A as indicated. HDAC2-V5 cotransfection induces

reporter gene expression in from all reporter gene constructs. Relative induction is highest with 4xκB-Luc. Graph shows relative luciferase units (RLU) derived of luciferase intensity after normalization to activity of cotransfected β-Gal. (**B**) Luciferase assay with 4xκB-Luc in HEK293T cells, as in Figure 1B but with catalytically inactive HDAC2^{H142A} included as control. Assay shows that HDAC2^{H142A} like HDAC2^{K462R} is not affecting reporter gene expression. Bar graph depicts mean ± sem of n=5 independent experiments. The values for pcDNA 3.1, HDAC2-V5 and HDAC2^{K462R}-V5 are a subset of those shown in Figure 1B. (**C**) Luciferase assay (4xκB-Luc) in HEK293T cells with testing induction of 4xκB-Luc with HDAC2 constructs having point mutations in the ΨKxE sumoylation consensus motif (VK^{462}EE in HDAC2). Notably, only mutant HDAC2^{E463A} in which the inert "x" position is mutated is able to increase reporter gene activity. Diagram show fold induction of mock transfected control, mean ± sem of n=3 independent experiments. (**D**) Summary of the HDAC2 mutants used, characterized by us and others. The catalytic activity of each mutant and the ability to become sumoylated is listed.

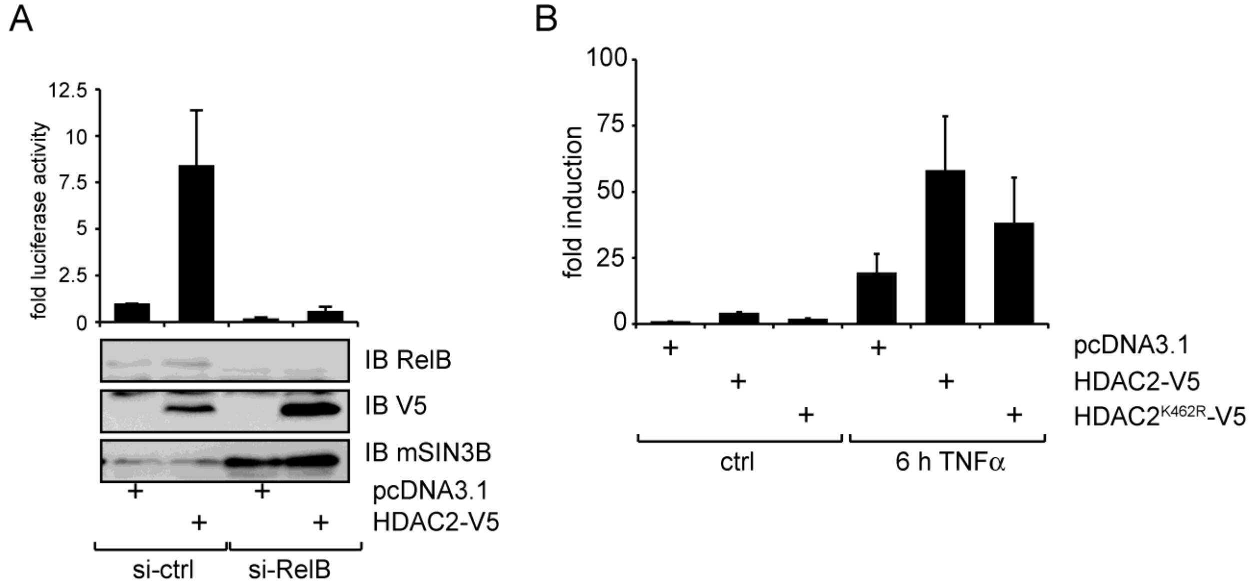

Supplementary Figure S2: (**A**) RKO cells transfected as in Figure 2A, knockdown of RelB diminishes HDAC2-caused NF-κB reporter gene induction; n=3. (**B**) RKO cells transfected as in Figure 1C with 4xκB-Luc assay together with HDAC2-V5 and HDAC2^{K462R}-V5. Cells were treated with 20 ng/ml TNFα for 6 h or left untreated for control prior to harvest. n=4. Induction of the reporter shows principally intact NF-κB signaling in these cells.

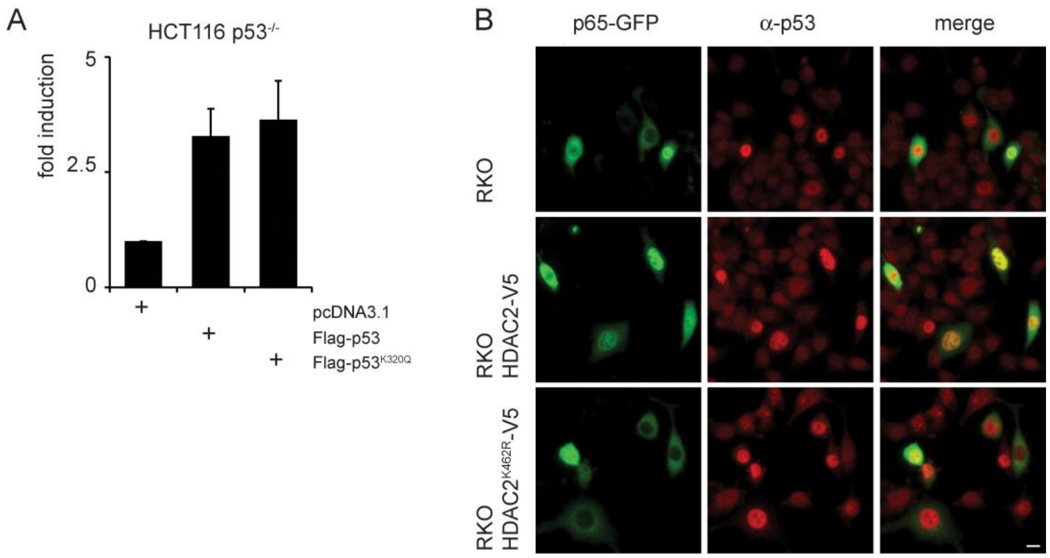

Supplementary Figure S3: (**A**) Luciferase assay with 4xκB-Luc in HCT116 $p53^{-/-}$ cells and reconstitution with either Flag-tagged wild-type p53 or a pseudo-acetylated $p53^{K320Q}$. n=4. (**B**) Representative image of RKO, RKO HDAC2-V5 and RKO $HDAC2^{K462R}$-V5 cells transiently transfected with p65-GFP as in Figure 3A, but with additional staining of endogenous p53. Bar = 10 µm.
